# SPI1 involvement in malignant melanoma pathogenesis by regulation of HK2 through the AKT1/mTOR pathway

**DOI:** 10.1111/jcmm.17844

**Published:** 2023-08-04

**Authors:** Chunlei Liu, Xiujuan Qiu, Jun Gao, Zhifan Gong, Xiaogang Zhou, Haichao Luo, Xuerui Geng

**Affiliations:** ^1^ Department of dermatology Xiangyang No.1 People's Hospital, Hubei University of Medicine Xiangyang China; ^2^ Department of oncology Xiangyang No.1 People's Hospital, Hubei University of Medicine Xiangyang China; ^3^ Department of general surgury Xiangyang No.1 People's Hospital, Hubei University of Medicine Xiangyang China

**Keywords:** AKT1/mTOR pathway, glycolysis, hexokinase 2 (HK2), melanoma, SPI‐1 proto‐oncogene (SPI1)

## Abstract

Spi‐1 proto‐oncogene (SPI1) plays a vital role in carcinogenesis. Our work aimed to investigate the potential regulatory mechanism of SPI1 in melanoma. The mRNA and protein levels were measured via qRT–PCR and Western blotting. Cell viability was assessed by CCK‐8 assay. The target relationship between SPI1 and hexokinase 2 (HK2) was determined using dual‐luciferase reporter detection. ChIP was conducted to confirm the targeted relationship between SPI1 and the HK2 promoter. Immunohistochemistry analysis was conducted to measure the positive cell number of SPI1 and HK2 in melanoma tissues. The cell migration abilities were determined using a wound healing assay. Glucose consumption, pyruvate dehydrogenase activity, lactate production and ATP levels were measured to assess glycolysis. SPI1 transcription in melanoma cells and tissues was dramatically higher than that in adjacent normal tissues and epidermal melanocyte HEMa‐LP, respectively. Knockdown of SPI1 restrained cell viability, metastasis and glycolysis in melanoma cells. SPI1 directly targeted HK2, and knockdown of SPI1 repressed HK2 expression. Overexpression of HK2 weakened the inhibitory effects of SPI1 knockdown on the viability, metastasis and glycolysis of melanoma cells. The serine–threonine kinase 1 (AKT1)/mammalian target of rapamycin (mTOR) axis is involved in melanoma progression. SPI1 knockdown restrained melanoma cell proliferation, metastasis and glycolysis by regulating the AKT1/mTOR pathway.

## 1 INTRODUCTION

Malignant melanoma is an aggressive cutaneous cancer. Although ultraviolet light is generally considered a factor in melanoma development, mechanical stimulation and genetic damage may influence melanoma progression.^1^ Melanoma accounts for only 1%–2% of all cancer cases but is responsible for approximately 95% of skin cancer deaths.^2^ There is little chance of melanoma metastasis from thin lesions.^3^ Therefore, it is very important to find tumour markers for the early diagnosis of skin cancer.

Spi‐1 proto‐oncogene (SPI1) is an oncogene specifically activated in acute murine erythroleukemias induced by the Friend spleen focus forming virus (SFFV), which is located in the p11.22 region of human chromosomes.^4^ As a transcription factor, SPI1 can be recruited by small nucleolar RNA host gene 16 (SNHG16) to regulate downstream gene expression, thus promoting the biological behaviour of cervical cancer cells.^5^ SPI1 has been shown to be associated with the disease progression of multiple malignant tumours, such as breast cancer,^6^ lung cancer,^7^ glioma^8^ and other types of neoplasia. SPI1 expression was markedly enhanced in gastric cancer, and high SPI1 expression was related to poor prognosis and tumour progression.^9^ We speculated that SPI1 might be a pathogenic factor for melanoma, and specific research studies on this subject have yet to be conducted.

Hexokinase 2 (HK2) is a rate‐limiting enzyme of glycolysis that is highly expressed in many human cancers and promotes proliferation, angiogenesis and glycolysis.^10^ HK2 has been proven to be involved in glycolysis in malignant melanoma cells.^11^ HK2 plays a vital role in regulating melanoma angiogenesis by promoting aerobic glycolysis and activating the p38‐mitogen‐activated protein kinase (MAPK) pathway,^12^ indicating that HK2 might be a candidate gene for melanoma, but specific studies are rarely reported. It is predicted by the hTFtarget database that SPI1 can bind to the HK2 promoter. The conserved binding sites of SPI1 were obtained from the JASPAR website (http://jaspar.genereg.net), and the potential binding sites of HK2 and SPI1 were predicted.

The AKT/mTOR signalling axis is involved in a wide range of cellular processes.^13^, ^14^ Enhancement of oxidative stress repressed the PI3K/AKT/mTOR axis in melanoma cells via disturbance of downstream protein phosphorylation.^15^ It has been demonstrated that knockdown of HK2 in renal cell carcinoma cells reversed the anti‐proliferative effects of betulin in an mTOR‐dependent manner.^16^ MTOR is a downstream molecule of AKT1, and the AKT/mTOR axis can meet the proliferation needs of gastric cancer cells via energy production activities (such as glycolysis).^17^ The regulation of melanoma proliferation, metastasis and glycolysis by HK2 through the AKT/mTOR signalling pathway has not been reported.

Here, we present the mechanism underlying the effect of SPI1 in regulating the biological behaviour of melanoma cells. In general, our work indicated that the transcription factor SPI1 promoted melanoma cell proliferation, metastasis and glycolysis by promoting HK2 expression and activating the AKT1/mTOR signalling pathway. This content has not yet been reported.

## 2 MATERIALS AND METHODS

### 2.1 Cell culture and tumour specimens

Melanoma tissues and normal adjacent tissues were collected from 30 patients with melanoma in the Xiangyang No.1 People's Hospital, Hubei University of Medicine, and all of the patients signed informed consent forms. This study was approved by Xiangyang No.1 People's Hospital, Hubei University of Medicine. Human melanoma cells A375, A2058, B16 and the invasive cells of choroidal melanoma (MUM2B) were provided by the Chinese Academy of Sciences (Shanghai, China), and epidermal melanocyte HEMa‐LP was obtained from Invitrogen. Cells were cultured in Dulbecco's Modified Eagle Medium (DMEM, Gibco) supplemented with 10% foetal bovine serum (FBS, Invitrogen) and 1% streptomycin at 37°C with 5% CO_2_ in a saturated humidified incubator.

### 2.2 Cell transfection

The specific short hairpin RNA (shRNA) against SPI1 (sh‐SPI1) and overexpression of HK2 (oe‐HK2) were purchased from Shanghai GenePharma company. Sh‐SPI1 was cloned into the lentiviral vector and then transfected into A375 and MUM2B cells. In addition, the vector, oe‐HK2 and oe‐NC were transfected into A375 cells through Lipofectamine 3000 reagent (Life Technologies Corporation) in line with the instructions.

### 2.3 Quantitative reverse transcript‐PCR (RT–qPCR)

Total RNA was extracted via TRIzol reagent (Sigma) in line with the manufacturer's protocol. SPI1 and HK2 were reverse transcribed using a reverse transcription kit (Takara). SPI1 and HK2 transcription was determined by SYBR Green Taq Mix (Takara). Glyceraldehyde‐3‐phosphate dehydrogenase (GAPDH) served as the internal reference. Primers are listed as follows: SPI1‐F, 5′‐CAGCTCTACCGCCACATGGA‐3′, and SPI1‐R, 5′‐TAGGAGACCTGGTGGCCAAGA‐3′, HK2‐F, 5′‐CTTCTTCACGGAGCTCAACC‐3′, and HK2‐R, 5′‐CTCCATCTCCTTGCGGAAC‐3′, GAPDH‐F, 5′‐CTGACATGCCGCCTGGAGA‐3′, and GAPDH‐R, 5′‐ATGTAGGCCATGAGGTCCAC‐3′.

### 2.4 Protein extraction and western blotting

The melanoma cells were lysed in cold RIPA reagent (Beyotime). After separation via SDS–PAGE, the proteins were transferred to PVDF membranes (Invitrogen). The membranes were blocked with 5% skim milk and probed with the following primary antibodies purchased from Abcam: anti‐SPI1 antibody (ab227835), anti‐HK2 antibody (ab209847), anti‐p‐AKT1 (ab81283), anti‐AKT1 (ab238477), anti‐p‐mTOR (ab109268) and anti‐mTOR (ab32028). Then, the membranes were incubated with anti‐rabbit IgG serum. After visualisation with enhanced chemiluminescence reagent, the density of the protein bands was analysed using ImageJ software.

### 2.5 Cell counting kit‐8 (CCK‐8) assay

Cell viability was determined by a CCK‐8 kit (Dojindo). Cells were plated into 96‐well plates at a density of 5 × 10^3^ cells/mL and cultured for 24, 48 and 72 h. CCK‐8 solution (10 μL) was added to each well, followed by incubation at 37°C for 2 h. The OD value at 450 nm was tested by a microwell reader (Analytik Jena AG).

#### 2.5.1 Wound healing assay

Cells cultured to confluence were scraped into a sharp edged cell‐free area. The transferred cells from the wound edge and the gap distance were counted under a microscope. The migration ability was assessed by the ratio of the gap distance at 48 h to that at 0 h.

#### 2.5.2 Immunohistochemical staining

The melanoma tissues and adjacent normal tissues were fixed using 4% formalin for 24 h and then cut by a slicer to 4 μm. The tissues were dried, dewaxed and rehydrated. Then, citrate buffer was applied, and tissues were autoclaved at 121°C for 90 s. After rinsing with PBS buffer, the tissues were blocked using goat serum (Gibco). The sections were treated with the following antibodies (all purchased from Abcam): anti‐SPI1 antibody (1:5000; ab227835), anti‐HK2 antibody (1:500, ab209847), anti‐p‐AKT1 (1:100, ab81283), anti‐AKT1 (1:500, ab238477), anti‐p‐mTOR (1:50 dilution, ab109268) and anti‐mTOR (1:400, ab32028). After rinsing with PBS buffer, the tissues were incubated with a detection system (OriGene Technologies). Subsequently, the sections were counterstained with haematoxylin at room temperature for 50 s.

#### 2.5.3 Glucose consumption determination

Glucose quantitation was assessed by a glucose assay kit in line with the manufacturer's protocols. DMEM high glucose medium (glucose concentration at 4500 mg/L) was applied as the experimental medium. A plate reader (Thermo Fisher Scientific) was used to measure absorbance at 570 nm.

#### 2.5.4 Measurement of lactate production

Lactate production was quantified by a lactate assay kit (BioVision). DMEM high glucose medium (glucose concentration at 4500 mg/L) was applied as the experimental medium. The lactate level was determined at 570 nm by a plate reader (Thermo Fisher Scientific).

#### 2.5.5 Detection of ATP

ATP concentration was detected via the ATP bioluminescent somatic cell assay kit (Sigma). The melanoma cells were collected, rinsed with PBS buffer, and resuspended in distilled water. After lysing by ATP releasing reagent, the cells were quantified using a Synergy HTX multimode reader (Bio‐Tek).

#### 2.5.6 Pyruvate dehydrogenase (PDH) activity

PDH activity was determined via a PDH activity assay kit (BioVision) in cell lysates.

### 2.6 Dual‐luciferase reporter assay

After insertion into the pmirGLO vector (GenePharma), HK2‐WT or HK2‐MUT and sh‐SPI1 or sh‐NC were introduced into melanoma cells. Lipofectamine 3000 reagent (Invitrogen) was applied, and the luciferase activity was monitored by a dual‐luciferase reporter assay system (Promega).

### 2.7 Chromatin immunoprecipitation (ChIP) assay

ChIP assays were carried out in line with the instructions of the MAGnify™ Chromatin Immunoprecipitation System (Invitrogen). The melanoma cells were fixed using 1% formaldehyde and sonicated to obtain DNA fragments. Appropriate anti‐SPI1 antibody (Abcam) was added, and anti‐H3 and normal IgG were used as positive and negative controls, respectively. After adding magnetic beads (Millipore) and vortexing, the complexes were digested with proteinase K, and DNA was purified using magnetic beads. The sites of interest were amplified by PCR.

### 2.8 Statistical analysis

All tests were conducted at least three times independently. SPSS 22.0 statistical software (SPSS Inc.) was applied for relevant data analysis. The data are indicated as the means ± S.D. Student's *t*‐test was adopted for pairwise comparisons, and multigroup comparisons were conducted using one‐way anova. *p* < 0.05 indicated a statistically significant difference.

## 3 RESULTS

### 3.1 SPI1 is highly expressed in melanoma tissues and cells

**FIGURE 1:**
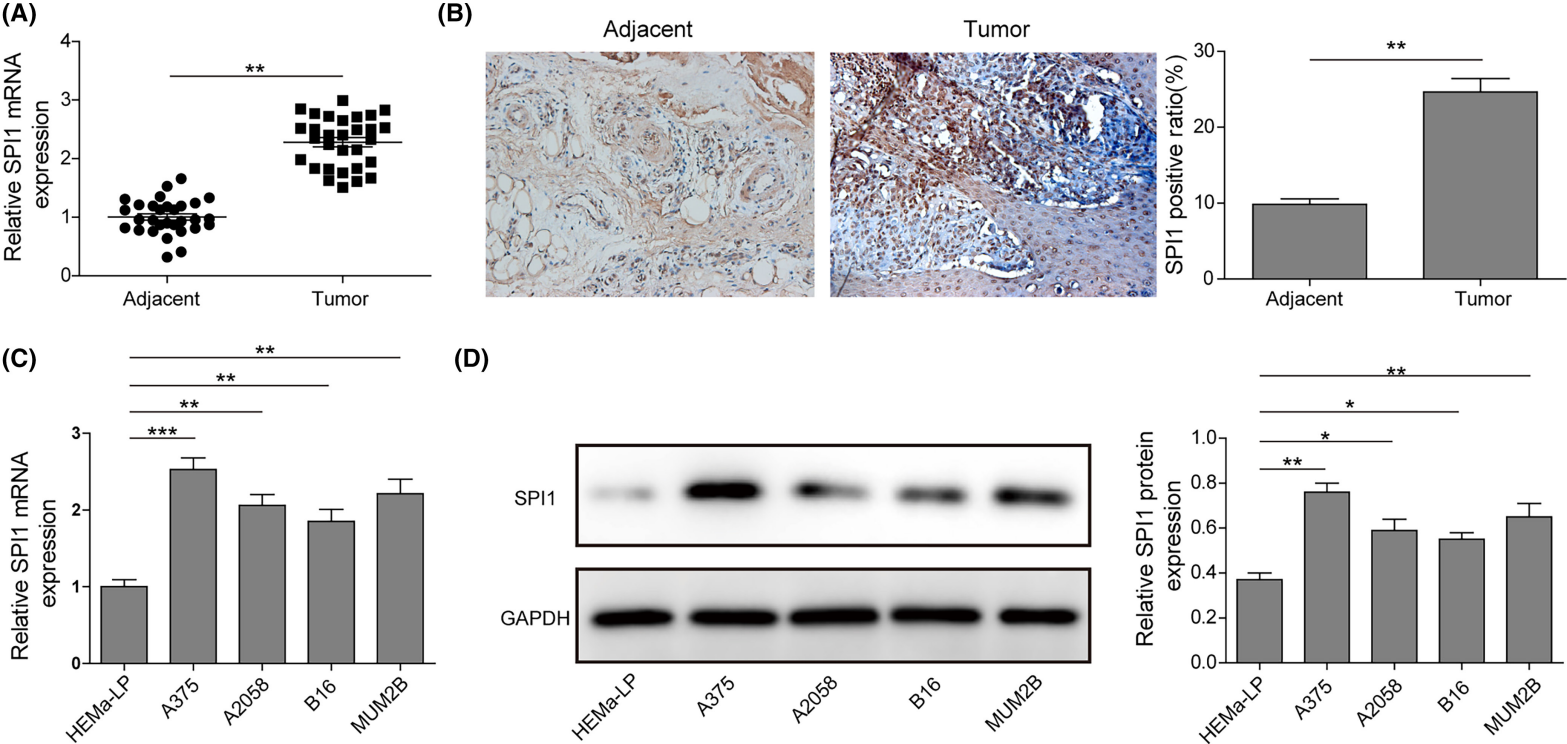
SPI1 is highly expressed in melanoma tissues and cells. (A) The mRNA expression of SPI1 in melanoma tissues and adjacent normal tissues was measured by qRT–PCR (*n* = 30). (B) The number of SPI1‐positive cells in melanoma tissues was measured by immunohistochemical analysis (*n* = 30). (C) The mRNA expression levels of SPI1 in melanoma cell Lines A375, A2058, B16 and MUM2B and epidermal melanocyte HEMa‐LP were determined by qRT–PCR assay (*n* = 3). (D) SPI1 protein expression in melanoma cells was monitored by western blotting (*n* = 3). The measurement data are expressed as the mean ± standard deviation. *p < 0.05, **p < 0.01, ***p < 0.001. All the above assays were executed at least three times.

qRT–PCR assays were conducted to determine the mRNA expression of SPI1 in 30 melanoma tissues and normal adjacent tissues. As shown in Figure 1A, SPI1 transcription in melanoma tissues was dramatically higher than that in adjacent normal tissues. Immunohistochemical detection showed that SPI1‐positive cell numbers in melanoma tissues were markedly enhanced (Figure 1B). Similarly, the mRNA expression levels of SPI1 in melanoma cell Lines A375, A2058, B16 and MUM2B were obviously higher than those in epidermal melanocyte HEMa‐LP and were highest in A375 and MUM2B cells (Figure 1C). Therefore, A375 and MUM2B cells were selected for subsequent experiments. Moreover, the SPI1 protein level was markedly increased in melanoma cells and was highest in A375 and MUM2B cells (Figure 1D). All the above data indicate that SPI1 might play a regulatory role in melanoma.

### 3.2 Knockdown of SPI1 suppresses melanoma cell proliferation, metastasis and glycolysis

**FIGURE 2:**
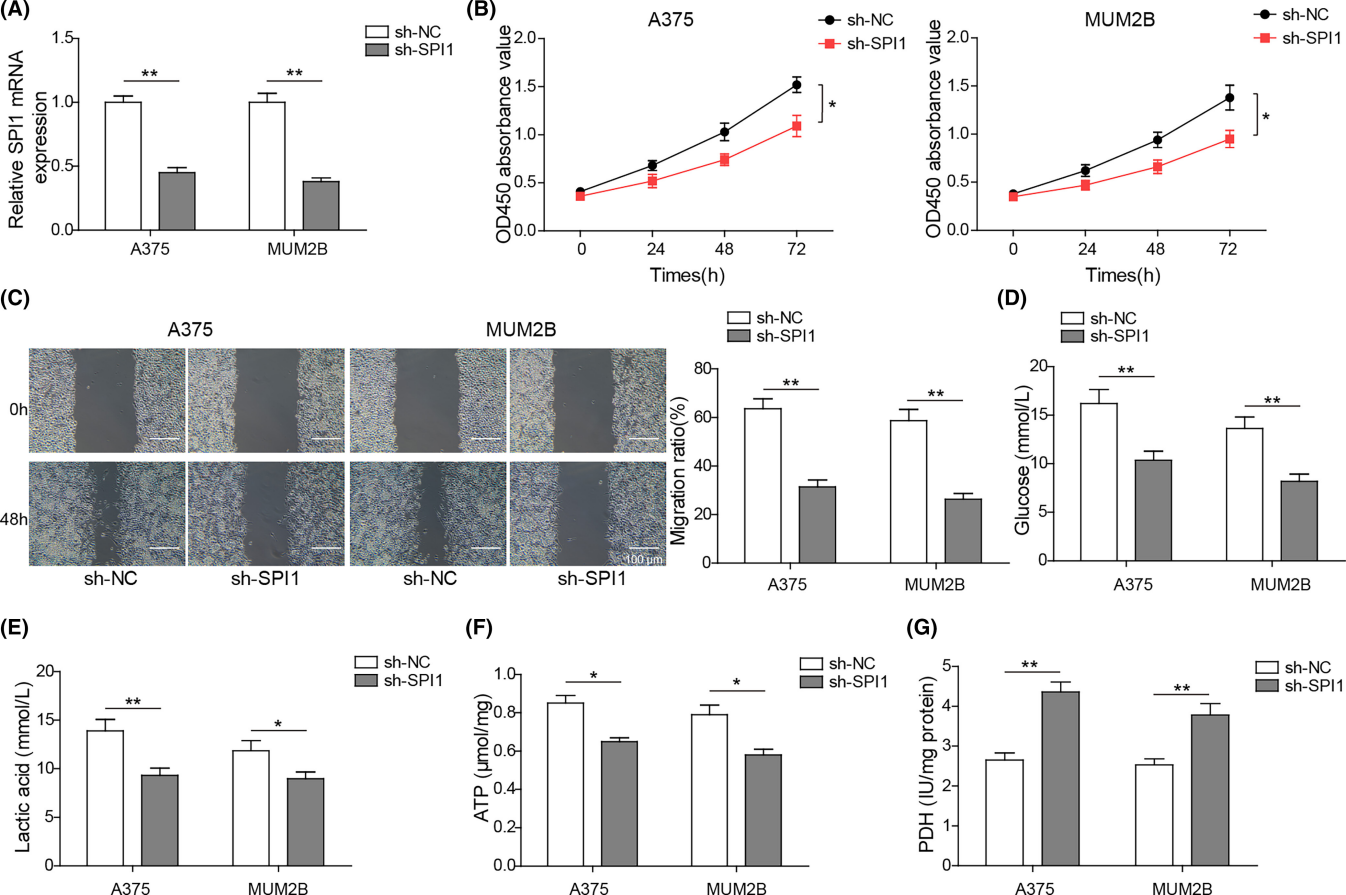
Knockdown of SPI1 suppresses melanoma cell proliferation, metastasis and glycolysis. A375 and MUM2B cell lines were transfected with sh‐SPI1 or sh‐NC. (A) The mRNA expression of SPI1 in A375 and MUM2B cells with SPI1 knockdown was measured via RT–qPCRqPCR (*n* = 3). (B) Cell viability was detected with a CCK‐8 assay (*n* = 3). (C) The cell migration abilities of A375 and MUM2B cells transfected with sh‐SPI1 or sh‐NC were determined using a wound healing assay (*n* = 3). (D‐G) Glycolysis of A375 and MUM2B cells transfected with sh‐SPI1 or sh‐NC was assessed by measuring glucose consumption (D), lactate production (E), ATP levels (F) and PDH activity (G) (*n* = 3). The measurement data are presented as the mean ± standard deviation. *p < 0.05, **p < 0.01. All the above assays were executed independently at least three times.

To verify the effects of SPI1 on the biological behaviour of melanoma cells, A375 and MUM2B cells were transfected with sh‐SPI1 or the negative control sh‐NC. As shown in Figure 2A, the SPI1 mRNA was downregulated when SPI1 was knocked down. Transfection with sh‐SPI1 suppressed the viability of A375 and MUM2B cells (Figure 2B). Moreover, the wound healing assay suggested that knockdown of SPI1 restrained cell migration, and the scratch width of the sh‐SPI1 group was wider than that of the sh‐NC group at 48 h (Figure 2C). As shown in Figure 2D,E, glucose consumption and lactate production were markedly lessened in A375 and MUM2B cells transfected with sh‐SPI1 relative to the control group. In addition, knockdown of SPI1 strikingly decreased ATP levels compared with those in the control group (Figure 2F). In addition, transfection with sh‐SPI1 overtly enhanced PDH activity (Figure 2G). These results suggest that knockdown of SPI1 can restrain cell proliferation, metastasis and glycolysis in melanoma cells.

### 3.3 SPI1 knockdown could inhibit HK2 expression by directly targeting the HK2 reporter

**FIGURE 3:**
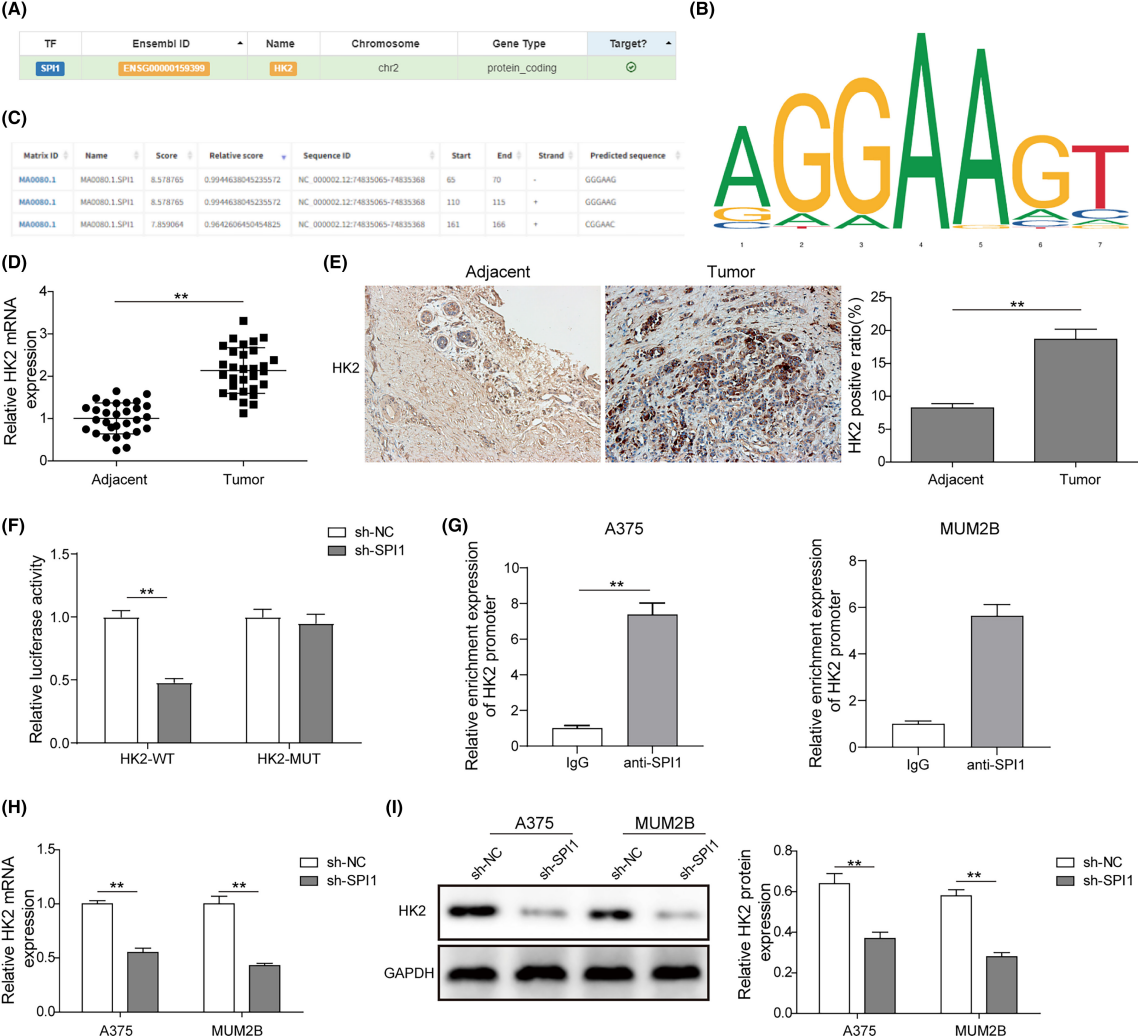
SPI1 knockdown inhibited HK2 expression by directly targeting the HK2 reporter. (A) The hTFtarget database was applied to predict the possible targets of SPI1. (B, C) Conserved binding sites of SPI1 (B) and the potential binding sites (C) between SPI1 and HK2 were predicted by the JASPAR database. (D) qRT–PCR analysis was performed to measure HK2 expression in melanoma tissues and adjacent normal tissues (*n* = 30). (E) Immunohistochemistry analysis was conducted to detect the number of HK2‐positive cells in melanoma tissues (*n* = 30). (F) The target relationship between SPI1 and HK2 was verified by dual‐luciferase reporter assay (*n* = 3). (G) A ChIP assay was performed to determine the targeting relationship between SPI1 and the HK2 promoter (*n* = 3). (H, I) HK2 mRNA (H) and protein (I) levels in A375 and MUM2B cells transfected with sh‐SPI1 were measured by qRT–PCR and western blotting (*n* = 3). The measurement data are expressed as the mean ± standard deviation. *p < 0.05, **p < 0.01. All the above assays were executed at least three times.

Subsequently, the hTFtarget database was used to predict the potential targets of SPI1. It was predicted that HK2 was the downstream factor of SPI1 (Figure 3A). As shown in Figure 3B,C, conserved binding sites of SPI1 and the possible binding sites between SPI1 and HK2 were predicted via the JASPAR database. qRT–PCR analysis indicated that HK2 transcription in melanoma tissues was dramatically elevated compared with that in adjacent normal tissues (Figure 3D). In addition, immunohistochemistry analysis showed that HK2‐positive cell numbers in melanoma tissues were significantly enhanced (Figure 3E). To verify the target relationship between SPI1 and HK2, a dual‐luciferase reporter assay was performed and showed that sh‐SPI1 prominently reduced the luciferase activity of the HK2 reporter (Figure 3F). Furthermore, ChIP assays showed that after the expression of SPI1 was inhibited, the enrichment of anti‐SPI1 in the HK2 promoter region decreased significantly (Figure 3G). In addition, the mRNA and protein levels of HK2 in A375 and MUM2B cells transfected with sh‐SPI1 were markedly reduced relative to those in sh‐NC‐transfected cells (Figure 3H, I). These data proved that SPI1 could bind to the HK2 reporter and that knockdown of SPI1 could repress HK2 expression.

### 3.4 Overexpression of HK2 weakens the inhibitory effect of sh‐SPI1 on the viability, metastasis and glycolysis of melanoma cells

**FIGURE 4:**
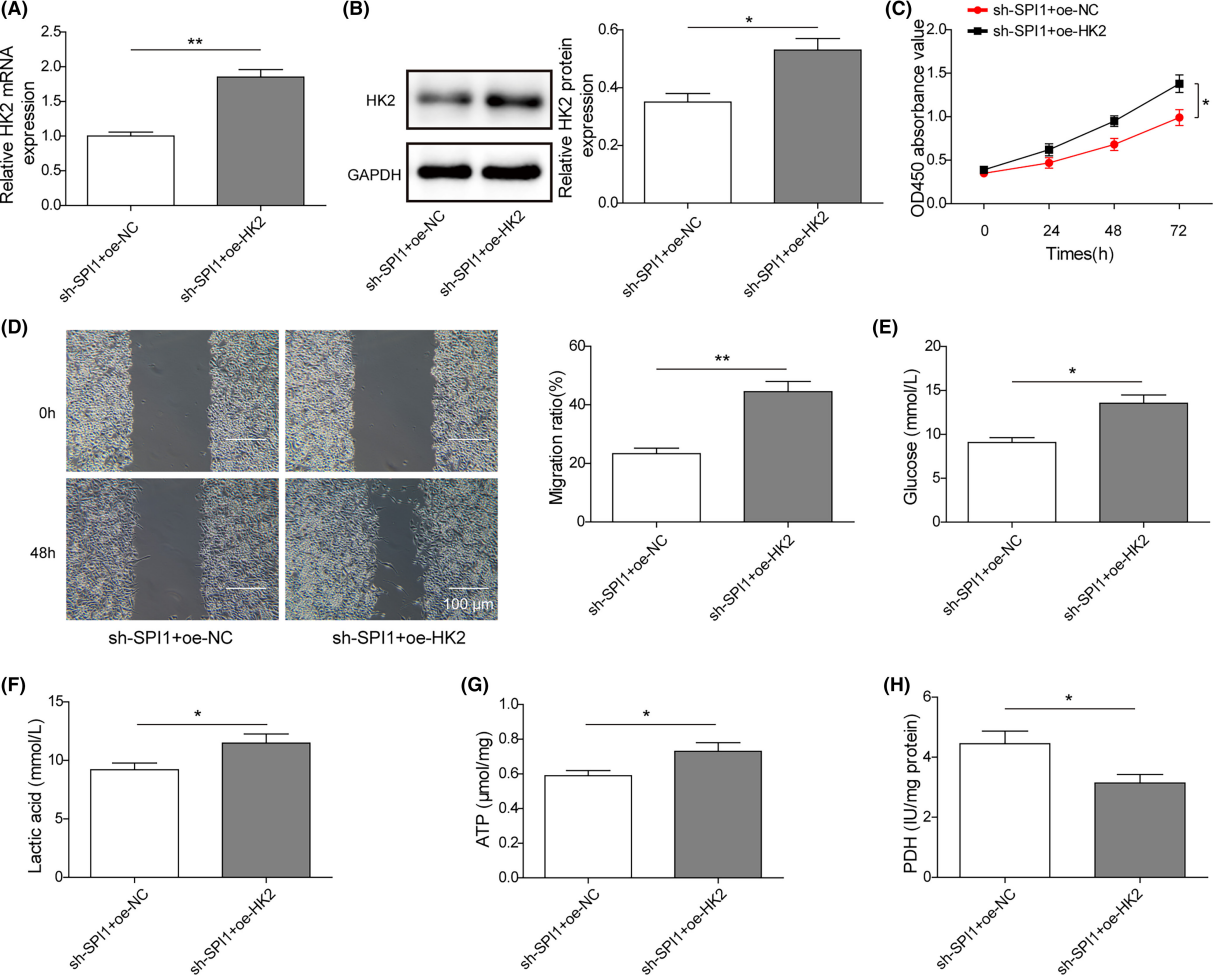
Overexpression of HK2 weakens the inhibitory effect of sh‐SPI1 on the proliferation, metastasis and glycolysis of melanoma cells. A375 cells were cotransfected with sh‐SPI1 and oe‐HK2. (A, B) HK2 mRNA (A) and protein (B) levels in A375 cells cotransfected with sh‐SPI1 and oe‐HK2 were measured (*n* = 3). (C) Cell viability was detected with a CCK‐8 assay (*n* = 3). (D) The cell migration abilities of A375 cells cotransfected with sh‐SPI1 and oe‐HK2 were determined using a wound healing assay (*n* = 3). (E‐H) Glycolysis of A375 and MUM2B cells cotransfected with sh‐SPI1 and oe‐HK2 was assessed by measuring glucose consumption (E), lactate production (F), ATP levels (G) and PDH activity (H) (*n* = 3). The measurement data are presented as the mean ± standard deviation. *p < 0.05, **p < 0.01. All the above assays were executed independently at least three times.

To illuminate whether SPI1 regulates the development of melanoma by targeting HK2, the A375 cell line with high activity was selected as the research object. First, SPI1 knockdown strikingly reduced the level of HK2 in A375 cells, whereas oe‐HK2 transfection abolished the decrease, and the HK2 mRNA level was obviously elevated (Figure 4A). Simultaneously, the HK2 protein level was decreased in cells of the sh‐SPI1 + oe‐NC group, while it was notably enhanced after co‐transfection of sh‐SPI1 and oe‐HK2 (Figure 4B). The CCK‐8 assay revealed that restrained melanoma cell viability due to sh‐SPI1 transfection was abrogated by HK2 overexpression (Figure 4C). Furthermore, introduction of oe‐HK2 weakened the depression of SPI1 knockdown on cell migration ability, and the scratch width of the sh‐SPI1 + oe‐HK2 group was narrower than that of the sh‐SPI1 + oe‐NC group at 48 h (Figure 4D). Additionally, the sh‐SPI1‐induced suppression of glycolysis was reversed by sh‐SPI1 and oe‐HK2 cotransfection. After transfection with sh‐SPI1 and further overexpression of HK2, glucose consumption was partially recovered (Figure 4E), lactate production was partly elevated (Figure 4F), ATP levels were significantly enhanced (Figure 4G) and PDH activity (Figure 4H) was partially reduced. In summary, these results demonstrated that SPI1 knockdown hindered melanoma cell viability, metastasis and glycolysis by sponging HK2

### 3.5 SPI1 regulates HK2 expression, thus promoting melanoma progression through the AKT1/mTOR pathway

**FIGURE 5:**
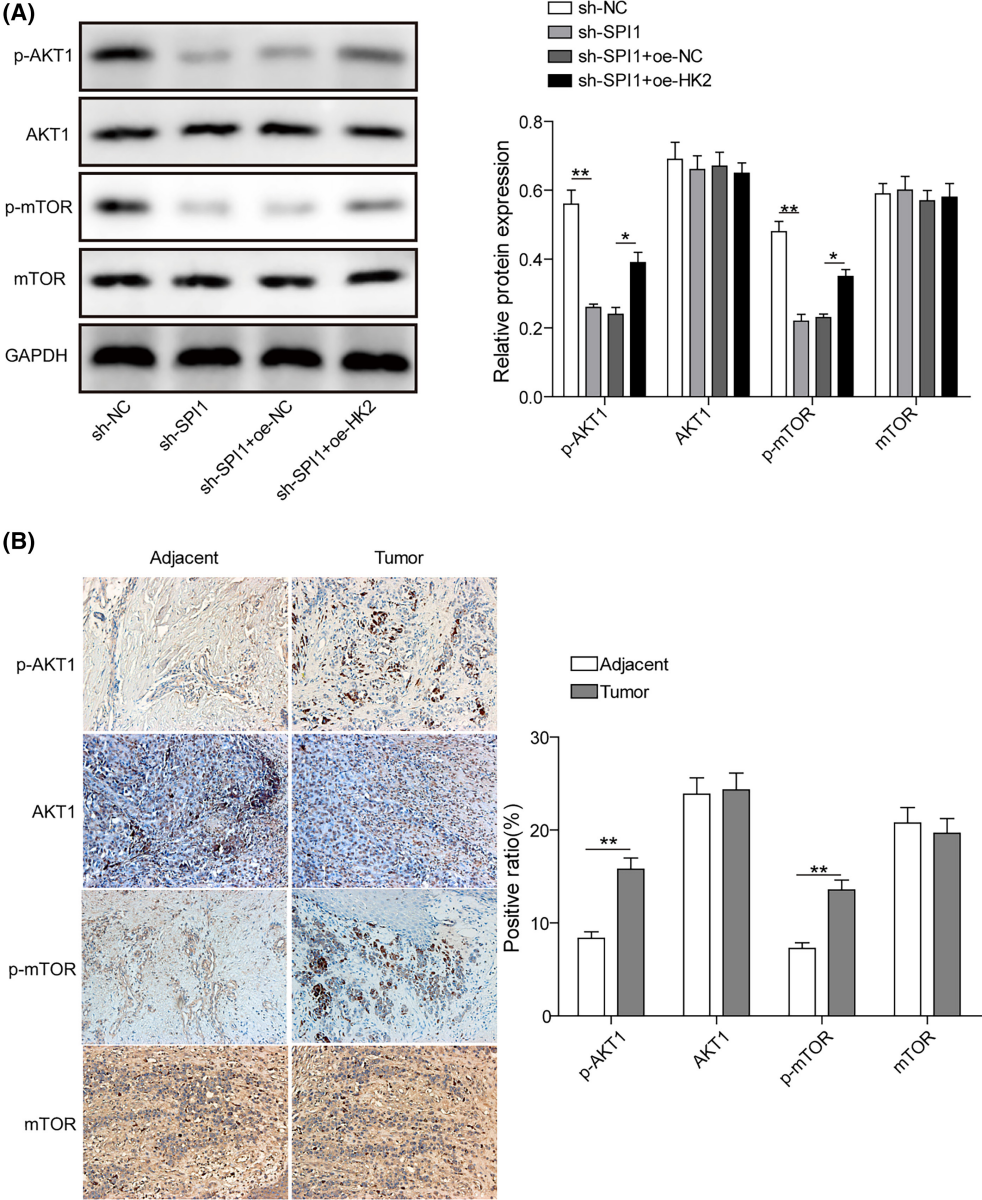
SPI1 regulates HK2 expression, thus promoting melanoma progression through the AKT1/mTOR pathway. (A) The protein levels of p‐AKT1 and p‐mTOR in A375 cells cotransfected with sh‐SPI1 and oe‐HK2 were measured (*n* = 3). (B) The protein levels of p‐AKT1, AKT1, p‐mTOR and mTOR in melanoma tissues were detected by immunohistochemistry assay (*n* = 30). The measurement data are presented as the mean ± standard deviation. *p < 0.05, **p < 0.01. All the above assays were executed independently at least three times.

To determine whether HK2 regulates melanoma cell progression through the AKT1/mTOR axis, further experiments were divided into the following groups: sh‐NC group, sh‐SPI1 group, sh‐SPI1 + oe‐NC group and sh‐SPI1 + oe‐HK2 group. Transfection with sh‐SPI1 and oe‐HK2 obviously enhanced p‐AKT1 and p‐mTOR levels compared with sh‐SPI1 and oe‐NC transfection (Figure 5A). The p‐AKT1, AKT1, p‐mTOR and mTOR protein levels in melanoma tissues were detected by immunohistochemistry assay, indicating that p‐AKT1 and p‐mTOR proteins were significantly increased in melanoma tissues (Figure 5B). These data validate that SPI1 regulates HK2 expression, thus modulating melanoma cell progression via the AKT1/mTOR axis.

## 4 DISCUSSION

In the present research, SPI1 was identified as a supposed oncogene in virus‐induced murine erythroleukemias.^18^ Activation of SPI1 has been shown to promote glycolysis in cancer cells, which in turn induces neutrophil N2 polarization through lactate, a glycolytic metabolite.^19^ SPI1 protein levels are higher in breast cancer tissues, and high SPI1 expression is correlated with the progression of breast cancer.^20^ Therefore, investigating the function of SPI1 in glycolysis is helpful for developing promising tumour therapeutic targets. Herein, we validated that SPI1 levels were strikingly elevated in melanoma cells and tissues and that SPI1 knockdown could restrain cell proliferation, metastasis and glycolysis in melanoma cells.

As SPI1 is a transcription factor that can modulate the expression of target genes by binding to specific DNA sequences of their promoters, the hTFtarget database was used to predict the potential targets of SPI1, and HK2 was selected as the downstream factor of SPI1. The dual‐luciferase reporter assay and ChIP assay verified the direct interaction of the SPI1 protein with the HK2 gene promoter. Thus, we chose HK2 as the research object for its cancer‐promoting effect in numerous malignancies.^21^ HK2 catalyses the first step in glucose metabolism, and HK2 is enriched in cancer cells, leading to a high glycolysis rate in tumours.^22^, ^23^ HK2 affects the invasion potential of pancreatic ductal adenocarcinoma (PDAC) cells by directly regulating glycolysis, and HK2 promotes the disease progression of PDAC by regulating lactate production.^24^ The findings in our study suggested that SPI1 interacted with HK2 and positively regulated its expression to promote melanoma progression. Furthermore, subsequent experiments indicated that HK2 overexpression weakened the inhibitory effect of SPI1 knockdown on cell viability, metastasis and glycolysis in melanoma cells.

Compelling research has revealed that PI3K/AKT/mTOR signalling could participate in promoting the osteosarcoma process by regulating a variety of target genes.^25^ The AKT1/mTOR axis might be involved in the disease progression of melanoma and modulated by minichromosome maintenance protein 7 (MCM7).^26^ In this study, p‐AKT1 and p‐mTOR levels were dramatically elevated in melanoma tissues, indicating that the AKT/mTOR signalling pathway might be involved in regulating melanoma progression. As demonstrated by Rao G, the glycolysis process could be regulated by the PI3K‐modulated AKT1/mTOR axis, thereby accelerating hypoxia‐inducible factor (HIF) activation to enhance angiogenesis and promote glucose uptake in cancer cells.^27^ PI3K/AKT/mTOR signalling is crucial to various aspects of tumour progression, such as cell proliferation, angiogenesis, tumorigenicity, invasion potential and survival.^28^ Herein, we verified that SPI1 regulated HK2 expression, thereby mediating the AKT1/mTOR signalling pathway and promoting melanoma cell proliferation, metastasis and glycolysis.

Altogether, our work elucidated that downregulation of SPI1 hampered cell proliferation, metastasis and glycolysis in melanoma cells by blocking the AKT1/mTOR axis by targeting HK2. This study may provide novel insight into potential therapeutic targets as well as the theoretical basis of the melanoma process. However, due to the limited sample size, these findings remain to be confirmed by a larger study. Future investigations may focus on the transcription factor‐DNA networks for SPI1 and the mechanisms involved in melanoma based on the findings of the present study.

## AUTHOR CONTRIBUTIONS

**Chunlei Liu:** Conceptualization (equal); methodology (equal); resources (equal); writing – original draft (equal); writing – review and editing (equal). **Xiujuan Qiu:** Conceptualization (equal); methodology (equal); resources (equal); writing – original draft (equal); writing – review and editing (equal). **Jun Gao:** Formal analysis (equal); investigation (equal); writing – review and editing (equal). **Zhifan Gong:** Investigation (equal); supervision (equal); writing – original draft (equal). **Xiaogang Zhou:** Investigation (equal); supervision (equal); writing – review and editing (equal). **Xuerui Geng:** Investigation (equal); software (equal); writing – review and editing (equal). **Haichao Luo:** Investigation (equal); supervision (equal); writing – review and editing (equal).

## CONFLICT OF INTEREST STATEMENT

The authors declare that they have no conflicts of interest.

## Data Availability

The raw data supporting the conclusions of this manuscript will be made available by the corresponding author(email: gengxuerui9240@126.com), without undue reservation, to any qualified researcher
